# Outcomes with Catheter-Directed Thrombolysis versus Mechanical Thrombectomy for Patients with Intermediate/High Risk Pulmonary Embolism: A Meta-Analysis of 24,090 Patients

**DOI:** 10.1007/s00408-026-00950-7

**Published:** 2026-11-10

**Authors:** Mohamed Saad Sayed, Doaa Mohamed Gebaly, Hasan Ilyas, Rawan Abdelhamed, Waleed Mohaned Rasheed, Hossam Hamdy, Lydia Melad, Giuseppe Andò, Toshiki Kuno, Islam Y. Elgendy

**Affiliations:** 1 Faculty of Medicine, Beni-Suef University, Ihnasya, Abdellatif Qasem Street, Beni Suef, Egypt; 2 Faculty of Medicine, Nahda University in Beni Suef (NUB), Beni Suef, Egypt; 3 Florida Atlantic University, Boca Raton, FL USA; 4 Faculty of Medicine, Tanta University, Tanta, Egypt; 5 Faculty of Medicine, Jordan University of Science and Technology, Irbid, Jordan; 6 Department of Clinical and Experimental Medicine, University of Messina, Messina, Italy; 7 Division of Cardiovascular Medicine, Knight Cardiovascular Institute, Oregon Health and Science University, Portland, OR USA; 8 Institute of Medicine, University of Tsukuba, Tsukuba, Japan; 9 Division of Cardiovascular Medicine, Gill Heart and Vascular Institute, University of Kentucky, Lexington, KY USA

**Keywords:** Pulmonary embolism, Catheter-directed medical thrombolysis, Mechanical thrombectomy, Large bore aspiration thrombectomy, Suction thrombectomy

## Abstract

**Background:**

Randomized trials comparing the outcomes with catheter-directed medical thrombolysis (CDT) versus mechanical thrombectomy (MT) for patients with intermediate/high risk pulmonary embolism (PE) are limited. This meta-analysis aimed to assess the clinical outcomes after CDT versus MT for patients with intermediate/high risk PE.

**Methods:**

Eligible studies compared CDT and MT for patients with intermediate/high risk PE underwent catheter-based therapies were retrieved from a comprehensive literature search for PubMed, Web of Science, Scopus, and Cochrane CENTRAL through June 2026. Outcomes included all-cause mortality, major bleeding, and the length of intensive care unit (ICU) stay. Summary estimates were analyzed using a random effects model.

**Results:**

A total of 18 studies (24,090 intermediate/high risk patients) were eligible for the analysis. CDT was associated with longer length of intensive care unit (ICU) stay (mean difference: 1.01 days, 95% confidence interval [CI] 0.87–1.15) compared with MT. While there was no statistical difference between the two groups regarding the risk of all-cause mortality (risk ratio [RR] 0.88, 95% CI 0.76–1.01, *p* = 0.068) and major bleeding (RR 1.07, 95% CI 0.74–1.55, *p* = 0.705). Similarly, the incidence of other clinical outcomes; all-cause readmission, PE-related readmission, blood transfusion rates, length of hospital stay, cardiac arrest, and stroke, were similar between the two groups.

**Conclusion:**

Among patients with intermediate/high risk PE treated with catheter-based therapies, there was no difference regarding the clinical outcomes between CDT and MT, but CDT was associated with a longer ICU stay. Larger, well designed randomized trials are needed to confirm these findings.

**Supplementary Information:**

The online version contains supplementary material available at https://doi.org/10.1007/s00408-026-00950-7.

## Introduction

Pulmonary embolism (PE) represents a significant contributor to the worldwide disease burden [1]. A variety of factors predispose individuals to PE, including recent surgery, malignancy, advanced age, and obesity, all of which are commonly encountered in modern clinical practice [2]. Consequently, despite ongoing advances in medical care, the risk of PE remains substantial, with an estimated incidence of approximately 115 cases per 100,000 individuals annually in the United States [3].

The treatment of PE has changed considerably over the years. Conventional therapies such as unfractionated heparin, vitamin K antagonists, and warfarin are increasingly being replaced by low-molecular-weight heparin (LMWH) and direct oral anticoagulants (DOACs), particularly in patients with low-risk disease [4]. In patients with high-risk PE, systemic thrombolysis has traditionally been used as an escalation strategy [5]. However, this approach is associated with an increased risk of intracranial bleeding [5]. These safety concerns have restricted its routine use and encouraged the exploration of alternative treatment therapies. Among these strategies, mechanical thrombectomy (MT) and catheter-directed thrombolysis (CDT), have emerged as minimally invasive treatment options, with their use increasing markedly over the past decade [6, 7].

Guideline recommendations for advanced reperfusion in acute PE vary by society and risk stratum. The 2019 ESC guidelines recommend against routine full-dose systemic thrombolysis in intermediate-high-risk PE but state that catheter-directed ttherapies and thrombolytic therapy may be considered in patients with clinical deterioration; in high-risk PE, catheter-directed thrombolysis should be considered as an alternative when systemic thrombolysis is contraindicated or has failed [8]. More recently, the 2026 AHA/ACC guidelines suggested that either CDT or MT might be considered for patients with acute PE without contraindications to thrombolysis and in whom advanced reperfusion therapy is indicated [9]. CDT works by delivering thrombolytic agents directly into the obstructed pulmonary arterial branches, allowing thrombus dissolution at lower doses compared with systemic thrombolysis [10]. In contrast, MT physically removes thrombi from the pulmonary circulation through aspiration or mechanical retrieval, aiming to re-establish pulmonary arterial flow and alleviate right ventricular strain in a less invasive manner than surgical embolectomy. This technique is generally performed without the use of thrombolytic agents, which may reduce bleeding risk [11].

The randomized controlled trials (RCTs) comparing CDT and MT are limited [12]. One retrospective observational study reported that MT was associated with a higher risk of major bleeding and all-cause readmission [13]. Conversely, the PEERLESS trial found no significant difference in major bleeding between the two approaches but reported a higher incidence of all-cause readmission and longer ICU stay after using CDT [12]. Similarly, a meta-analysis comparing MT and CDT found no significant differences in all-cause mortality, readmission, or major bleeding between the two modalities [14]. Hence, several additional studies have been published recently, which were not included in earlier analyses [15–19]. Therefore, an updated meta-analysis is needed to synthesize up-to-date analysis for comparison between CDT and MT among patients with intermediate/high risk PE underwent catheter-based therapies.

## Methods

This meta-analysis was performed following the Preferred Reporting Items for Systematic Reviews and Meta-Analyses (PRISMA) guidelines [20]. The study was also conducted in accordance with the recommendations outlined in the Cochrane Handbook for Systematic Reviews [21]. The protocol for this meta-analysis was prospectively designed and registered to the international prospective register of systematic reviews (PROSPERO) with an ID: CRD420261445909. Ethical approval was not required because the analysis was based solely on data from previously published studies.

### Data Sources and Literature Search

A comprehensive literature search was conducted through four databases: PubMed, Cochrane Central, Scopus, and Web of Science from inception to June 2026 to identify studies comparing CDT and MT in adults with intermediate/high risk PE underwent catheter-based therapies. The search strategy incorporated both controlled vocabulary and relevant keywords related to CDT, MT, and acute PE. The complete search strategy is provided in Table S1. Additionally, the reference lists of eligible articles and previously published meta-analyses were manually screened to identify any additional relevant studies that may have been missed during the initial search. To cover the grey literature, we searched on Google Scholar and Clinicaltrials.gov manually using the previous search strategy.

### Eligibility Criteria and Study Selection

Studies were considered eligible if they met the following criteria: (a) randomized controlled trials (RCTs), cohort studies, or any comparative observational study; (b) included adult patients diagnosed with intermediate/high risk PE underwent catheter-based therapies; and (c) compared clinical outcomes with CDT vs. MT.

Studies were excluded if they met any of the following criteria: (a) included patients younger than 18 years of age; (b) involved overlapping populations, defined by studies originating from the same institutions with similar recruitment periods; (c) evaluated populations outside the scope of interest; (d) were republished studies or duplicate reports; (e) study protocols without reported results; (f) publication types such as reviews, abstracts, case reports, case series, background articles, expert opinions, or in vivo/in vitro studies; (g) duplicate data derived from the same clinical trial; or (h) studies lacking a comparator group.

Study selection was performed independently by two investigators (D.M.G., and R.A.) in a two-step screening process. In the first phase, titles and abstracts of all retrieved records were screened for potential eligibility. In the second phase, the full texts of potentially relevant studies were reviewed to determine final inclusion. Any disagreements between reviewers were resolved through discussion, and when necessary, consultation with a third investigator (M.S.S.) to reach a consensus.

### Data Extraction and Outcomes

Data extraction was independently performed by two reviewers (D.M.G., and R.A.) using a standardized data collection form. Extracted variables included study characteristics, inclusion criteria, devices used, PE severity, follow-up duration, baseline patient characteristics, and reported clinical outcomes. The outcome of interest was all-cause mortality. Other outcomes include major bleeding, length of intensive care unit (ICU) stay, all-cause readmission, PE-related readmission, blood transfusion rates, length of hospital stay, cardiac arrest, and stroke.

### Quality Assessment

The risk of bias for RCTs was evaluated using the revised five-domain Cochrane Risk of Bias tool (RoB 2.0) [22], which assesses domains including the randomization process, allocation concealment, blinding, attrition, selective outcome reporting, and other potential sources of bias. Observational studies were assessed using seven-domain risk of bias in non-randomized studies-of interventions (ROBINS-I) tool [23]. Two reviewers (D.M.G., and R.A.) assessed the quality of included studies, and any disputes were resolved with a third reviewer (M.S.S.).

### Statistical Analysis

The events and totals for the dichotomous outcomes were obtained to calculate risk ratios (RRs) with the corresponding 95% confidence interval (CI), while the mean and standard deviation was obtained for the continuous data to calculate the mean difference (MD) with the corresponding 95% CI. Random effects model restricted likelihood ratio (REML) was used for the primary analysis. Statistical heterogeneity was observed using I^2^ statistics according to Cochrane Handbook which stated that I^2^ <50% considered a low heterogeneity while I^2^ >50% considered a high heterogeneity and needs clarifications [21]. Sensitivity analysis leaving one study out at each time of analysis was performed for the outcomes with a high heterogeneity to address the source of heterogeneity. Further sensitivity analysis was performed including the studies that used the EKOS system for CDT. Subgroup analyses were planned to be performed based on follow-up period: in-hospital, ≤ 30 days, ≤ 1 year and 5 years, number of centers: single-center and multicenter (more than two centers), and study design: observational and RCT. The P_interaction_ <0.05 was considered significant with an interaction between groups. Random effects meta-regression analysis using study covariates such as age, male sex, body mass index (BMI), smoking, and diabetes mellitus (DM). The Egger’s regression test and Begg’s rank correlation test were conducted to detect the publication bias. All analyses were conducted using R software [24].

## Results

### Study Selection and Characteristics

**Fig. 1:**
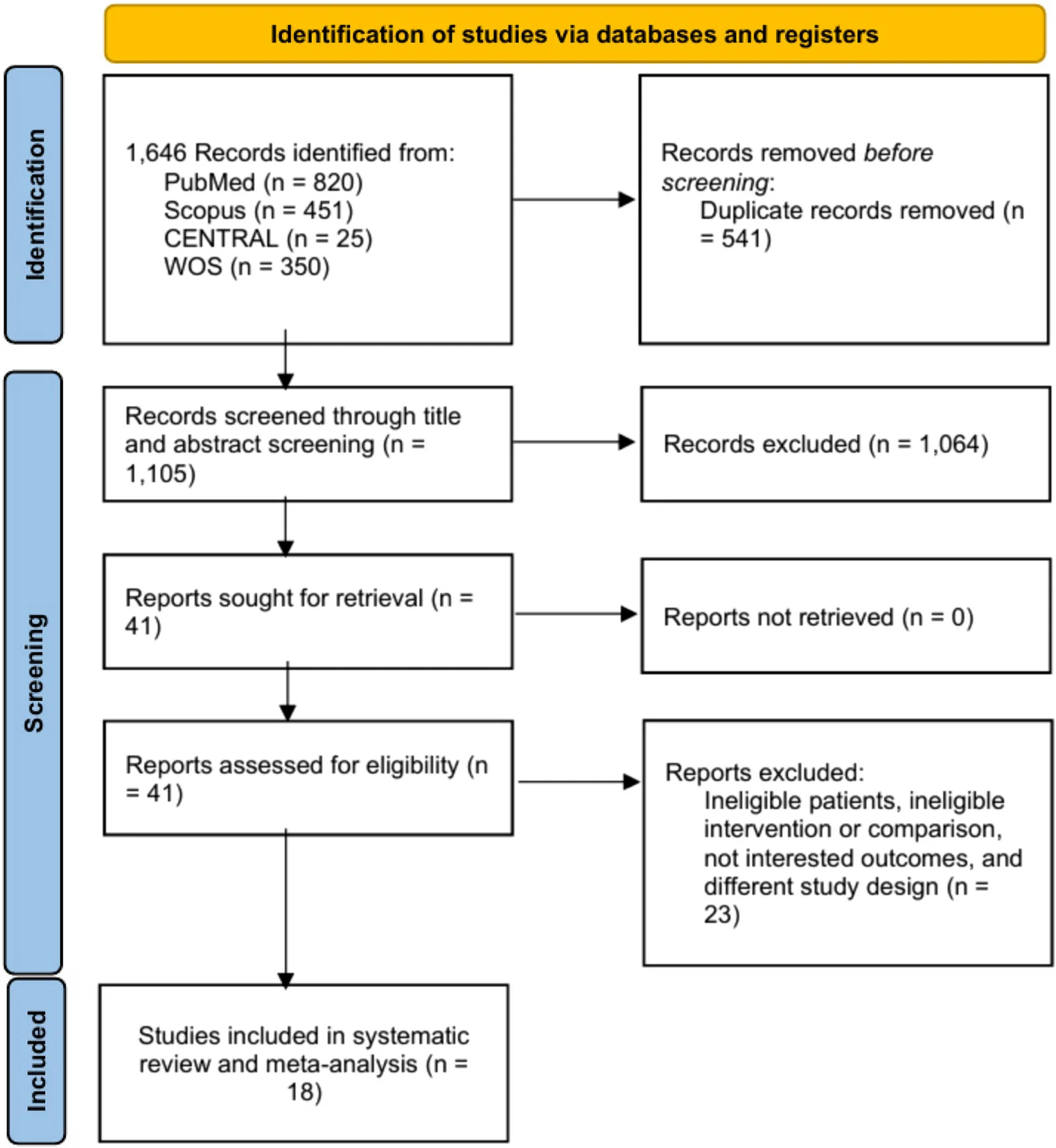
PRISMA Flowchart for study selection process from search to included studies

The selection process yielded 18 studies [12, 13, 15–19, 25–35] that were eligible for the analysis (Fig. 1). A total of 24,090 patients were analyzed from the included studies. Only one study was an RCT [12], while the other 17 studies [13, 15–19, 25–35] were cohort studies (all were retrospective). The weighted mean follow-up was 8.07 months. The follow-up period ranged from one day to 60 months, with 5 studies followed up to 1 month. Regarding the centers of studies, 8 studies were single center [15, 18, 25, 26, 29, 30, 32, 33], while 10 studies [12, 13, 16, 17, 19, 27, 28, 31, 34, 35] were multicenter. A total of 13 studies were conducted in the USA [12, 13, 15–17, 19, 25–27, 29–32], in addition to one study conducted in 19 countries [34], but mainly was in the USA. While two studies in Germany [28, 35], one in China [18], and one in Poland [33]. Summary of baseline characteristics are shown in Tables 1 and 2. Additionally, Table S2 is used to summarize the CDT device, drug and dose used, and MT device.

**Table 1:** Summary of included studies

Study ID	Study design	Centers	Country	Sample size	CDT Device	CDT modality	CDT drug	MT	PE patients	Diagnosis of PE	FU, months
Demaree et al. 2026 [15]	Observational study	Single center	USA	461	either the Unifuse (Angiodynamics) or Cragg-McNamara (Medtronic) conventional infusion catheters	Ultrasound	rtPA at 0.5–1 mg/h	A balloon-tipped or pigtail catheter.	Intermediate and high-risk PE	CT angiography or V/Q scan	1 month
Shammas et al. 2025 [17]	Observational study	Multicenter	USA	97	The EKOS Endovascular System (Boston Scientific)	Ultrasound	rtPA at 0.5–1 mg/h	The FlowTriever (Inari Medical).	Intermediate risk PE	CT angiography	1 month
Zhang et al. 2025 [18]	Observational study	Single center	China	58	Using 1 or 2 multiple-sided hole infusion catheters (Multi-Sideport; Cook, Bloomington, IN, USA)	Ultrasound	Urokinase at a dosage of 200,000–800,000 IU/day.	The Indigo Catheter (8 Fr) (Penumbra)	Intermediate and high-risk PE	CT angiography and D-dimer	24 months
Watanabe et al. 2025 [19]	Observational study	Multicenter	USA	719	-	Ultrasound	-	-	High-risk PE	CT angiography	12 months
Rosenzveig et al. 2025 [16]	Observational study	Multicenter	USA	4018	-	-	-	-	Intermediate and high-risk PE	CT angiography	12 months
Pinsdorf et al. 2025 [28]	Observational study	Multicenter	Germany	26	The EKOS Endovascular System (Boston Scientific)	Ultrasound	Alteplase at a rate of 1 mg/h for 6–12 h, alongside UFH anticoagulation maintaining a PTT of 50–70 s.	The FlowTriever System (Inari Medical)	Intermediate and high-risk PE	CT angiography	3 months
Majmundar et al. 2025 [13]	Observational study	Multicenter	USA	14,731	-	-	-	-	Intermediate and high-risk PE	CT angiography	9 months
Kattih et al. 2024 [32]	Observational study	Single center	USA	123	-	-	-	-	Intermediate and high-risk PE	Clinical diagnosis triggering PERT activation	12 months
Al-Terki et al. 2024 [35]	Observational study	Multicenter	Germany	95	The EKOS Endovascular System (Boston Scientific)	Ultrasound	1 mg/h alteplase for 6 h in parallel with an anticoagulation with UFH with a PTT of 60–80 s.	The LBT system (Inari Medical)	Intermediate and high-risk PE	Multislice computed tomography (MSCT) of lungs	1 day
Al Soueidy et al. 2024 [25]	Observational study	Single center	USA	189	The EKOS Endovascular System (Boston Scientific)	Ultrasound	tPA within the pulmonary arteries, typically 1 mg/hour per catheter, for a total dose ranging from 12 to 24 mg.	Penumbra	Intermediate and high-risk PE	CTPA or echocardiogram	2–3 days post-procedure
Jaber et al. 2024 [12]	RCT	Multicenter	USA	550	The EKOS Endovascular System (Boston Scientific)	Ultrasound	-	FlowTriever System (Inari Medical, Irvine, CA).	Intermediate risk PE	CTPA or echocardiogram	1 month
Szmyt et al. 2024^33^	Observational study	Single center	Poland	42	-	-	Alteplase at a dose of 1 mg/h/catheter for 8 h with initial bolts of 2 mg/catheter	-	Intermediate and high-risk PE	CTPA or troponin levels or TTE	1.5 days
Tsugakoshi et al. 2024 [34]	Observational study	Multicenter	19 countries mainly USA	2204	**-**	**-**	-	**-**	Intermediate and high-risk PE	CT angiography	60 months
Feroze et al. 2023 [27]	Observational study	Multicenter	USA	147	The EKOS Endovascular System (Boston Scientific)	Ultrasound	-	The Inari FlowTriever thrombectomy catheter (Inari Medical)	Intermediate and high-risk PE	CT angiography	12 months
Tomaszek et al. 2023 [30]	Observational study	Single center	USA	48	-	-	tPA drip rate of 0.5–1 mg/h	The FlowTriever System (Inari Medical, Irvine, CA)	Intermediate and high-risk PE	CT angiography	1 day
Inci et al. 2022 [31]	Observational study	Multicenter	USA	458	The Ekosonic (Boston Scientific), Unifuse (Angiodynamics), or Cragg-McNamarra (Medtronic) catheters	Fluoroscopy	rTPA	The FlowTriever System (Inari Medical, Irvine, CA)	Intermediate and high-risk PE	CT or direct angiography	1 month
Graif et al. 2020 [29]	Observational study	Single center	USA	52	Cook Medical, Bloomington, Indiana), UniFuse multiple side-hole infusion catheters (AngioDynamics, Inc, Latham, New York), and EkoSonic Endovascular System catheters (Boston Scientific, Marlborough, Massachusetts).	Ultrasound	tPA	The FlowTriever (Inari Medical, Irvine, California)	Intermediate and high-risk PE	CT angiography	1 month
Avgerinos et al. 2018 [26]	Observational study	Single center	USA	72	The Eko-Sonic catheter (EKOS Corp, Bothell, Wash)	Ultrasound	2 to 4 mg of rTPA is given when needed, followed by lysis initiation at a rate of 0.5 to 1.0 mg/h.	5 F Cragg-McNamara [Boston Scientific, Marlborough, Mass] or UniFuse [AngioDynamics, Latham, NY]	Intermediate and high-risk PE	CT angiography	---

*CDT* catheter-directed thrombolysis, *MT* mechanical thrombectomy, *TTE* transthoracic echocardiography, *CTPA* computed tomography pulmonary angiography, *UFH* unfractionated heparin, *rTPA* Tissue plasminogen activator

**Table 2:** Baseline characteristics of patients through included studies

Study ID	Age, years mean (SD)		Male, n (%)		BMI, mean (SD)		Pulmonary disease, n (%)		Smoking, *n* (%)		Diabetes, n (%)	
	MT	CDT	MT	CDT	MT	CDT	MT	CDT	MT	CDT	MT	CDT
Demaree et al. 2026 [15]	62.0 (16.7)	57.4 (16.1)	163 (52.9)	79 (51.6)	34.9 (10.5)	35.8 (9.0)	-	-	72 (23.4)	29 (18.9)	76 (24.7)	33 (21.6)
Shammas et al. 2025 [17]	66.53 (62.28)	62.28 (14.45)	26(60.5)	34 (63)	34.3 (9.4)	34.3 (9.6)	-	-	16 (37.3)	22 (40.7)	16 (37.2)	14 (25.9)
Zhang et al. 2025 [18]	67 (14.9)	66.8 (12.9)	13 (44.8)	17 (58.6)	-	-	-	-	3 (10.3)	5 (17.2)	4 (13.8)	5 (17.2)
Watanabe et al. 2025 [19]	74 (7.4)	74 (8.15)	105 (43.9)	196(41)	-	-	30(12.7)	58(12.1)	-	-	80 (33.5)	154 (32.2)
Rosenzveig et al. 2025 [16]	59.9	60.7	2278 (54.9)	1176 (58.3)	-	-	-	-	-	-	-	-
Pinsdorf et al. 2025 [28]	60.5 (14.2)	61.8 (16)	8 (66.7)	9 (64.3)	30.5 (4.9)	34 (10.6)	2 (16.7)	3 (21.4)	-	-	0 (0)	5 (35.7)
Majmundar et al. 2025 [13]	61.5 (15.2)	61.7 (15.1)	3754 (50.9)	3803 (51.7)	-	-	1554 (20.5)	1520 (20.5)	1,694 (22.9)	1,739 (23.3)	2,003 (26.8)	1,968 (26.6)
Kattih et al. 2024 [32]	-	-	-	-	-	-	-	-	-	-	-	-
Al-Terki et al. 2024 [35]	68 (10.7)	66 (14.7)	17 (65)	31 (45)	29.7 (4.6)	29.2 (6.6)	1 (4)	14 (20)	-	-	-	-
Al Soueidy et al. 2024 [25]	61.9 (14.6)	59.8 (14.0)	37 (52.9)	67 (56.3)	33.2 (8.4)	34.2 (9.2)	11 (15.7)	18 (15.1)	26 (37.1)	39 (32.8)	14 (20.0)	30 (25.2)
Jaber et al. 2024 [12]	63.7 (13)	61.2 (14.8)	149(54.4)	142(51.4)	34.5 (8.6)	36.3 (9.4)	-	-	-	-	-	-
Szmyt et al. 2024 [33]	58.8 (14.9)	54.8 (14.7)	8(38.1)	12(57.14)	29.2 (4.6)	29.4 (5.7)	-	2(9.5)	-	-	2 (9.5)	4(19)
Tsugakoshi et al. 2024 [34]	59.1(15.2)	58.5(15.1)	577(52.4)	577 (52.4)	-	-	116 (10.5)	118 (10.7)	198 (18.0)	212 (19.2)	297 (27.0)	288 (26.1)
Feroze et al. 2023 [27]	64 (15.6)	59 (14.8)	53 (54.6)	23 (46)	-	-	24 (24.7)	18 (36.0)	52 (53.6)	30 (60)	-	-
Tomaszek et al. 2023 [30]	64.7 (11.5)	58.8 (12.2)	11(35.5)	7(41.2)	-	-	6 (19.4)	0 (0)	-	-	-	-
Inci et al. 2022 [31]	59 (1.1)	56 (0.9)	80 (42)	135 (51)	34 (0.6)	35 (0.5)	27 (14.)	59 (22)	-	-	42 ( 22)	51 (19)
Graif et al. 2020 [29]	60.2 (17.1)	59.7 (14.2)	12 (46.2)	12 (46.2)	34.4 (10.5)	38.6 (10.2)	7 (26.9)	3 (11.5)	3 (11.5)	2 (7.7)	7 (26.9)	5(19.2)
Avgerinos et al. 2018 [26]	64.1 (14.1)	63.5 (14.2)	9 (50)	22 (40.7)	-	-	3 (16.7)	10 (18.5)	-	-	-	-

*CDT* catheter-directed thrombolysis, *MT* mechanical thrombectomy

### Quality Assessment

A total of 17 studies [13, 15–19, 25–35] were assessed by ROBINS-I while one RCT was assessed by ROB 2.0 [12]. Six studies [16, 19, 27, 28, 30, 32] were assessed as moderate at overall quality assessment, while 11 studies [12, 13, 15, 17, 18, 25, 26, 29, 31, 33, 35] were at low risk of bias. The only RCT [12] was assessed as low risk of bias. Figure S1 shows the quality assessment of included studies; either ROB 2.0 or ROBIN-I.

### Outcomes

#### Outcomes of Interest

##### All-Cause Mortality

**Fig. 2:**
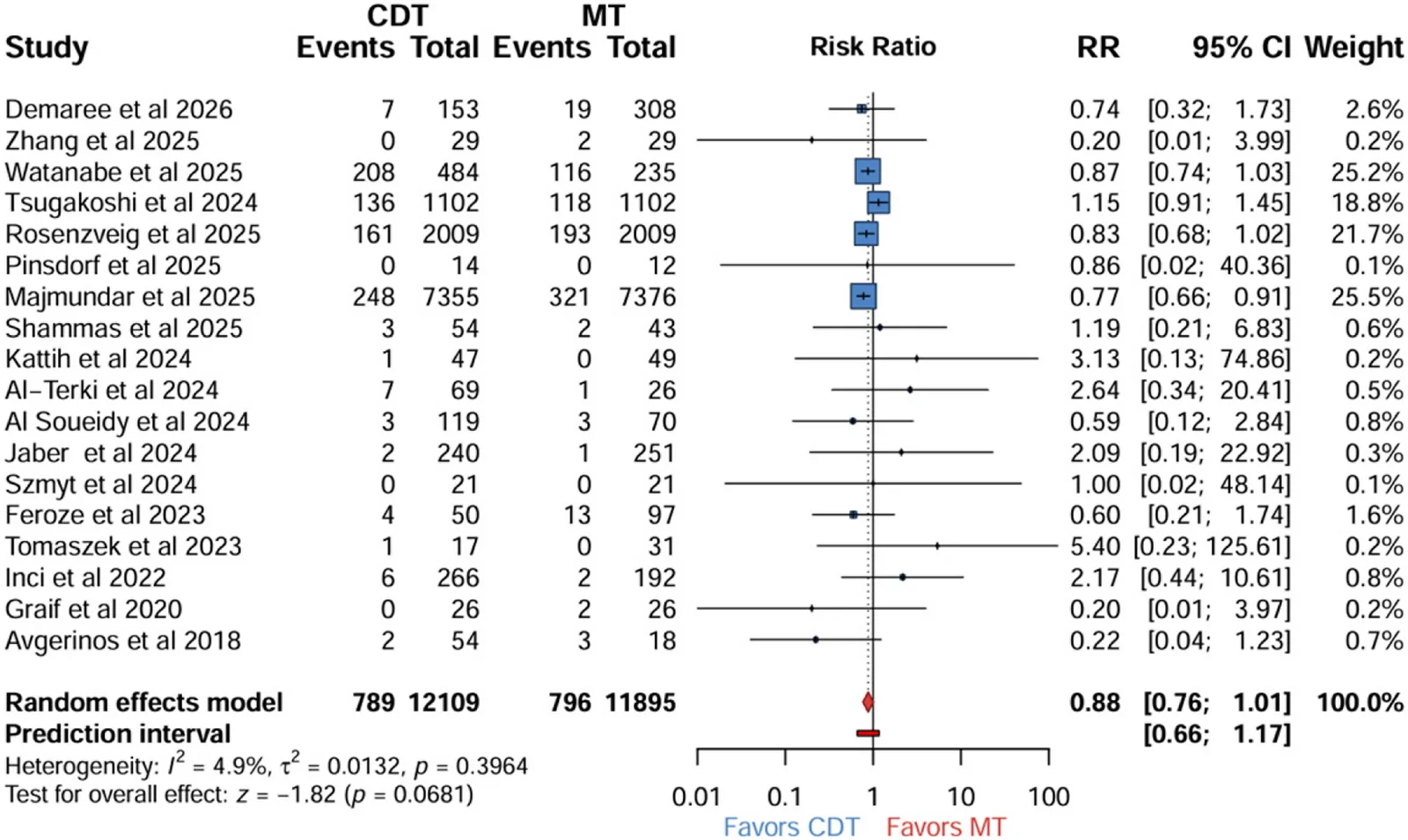
Forest plot for all-cause mortality. CDT, catheter directed thrombolysis; MT, mechanical thrombectomy; RR, risk ratio

All studies reported the risk of all-cause mortality. Pooled estimate of 24,004 patients showed no statistical difference in the incidence of all-cause mortality with CDT compared with MT (RR 0.88, 95% CI 0.76–1.01, *p* = 0.068, I^2^ = 4.9%, Fig. 2). The prediction interval ranged from 0.66 to 1.17. A sensitivity analysis including the studies that used the EKOS system for CDT revealed no significant difference between CDT and MT (RR 0.85, 95% CI 0.47–1.53, *p* = 0.58, I^2^ = 0.0%) (Figure S2). The subgroup analysis based on follow-up period revealed a significant lower incidence of in-hospital all-cause mortality after CDT compared with MT (RR 0.80, 95% CI 0.70–0.91, I^2^ = 0.0%); but there was no significant difference in the incidence of ≤ 30 days, ≤ 1 year and 5 years all-cause mortality between two groups with a significant subgroup difference (P_interaction_ = 0.003) (Figure S3). Subgroup analyses revealed similar risks of all-cause mortality between the two groups without significant interaction based on the study design: observational versus RCT (P_interaction_ = 0.61) and centers: single-center versus multicenter (P_interaction_ = 0.86) (Figures S4-5).

Random-effects meta-regression analysis revealed no significant association between mortality risk and study covariates such as age (*p* = 0.53), male sex (*p* = 0.34), BMI (*p* = 0.39), smoking (*p* = 0.48), and DM (*p* = 0.69). Additionally, there was no publication bias in the analysis, confirmed with an insignificant Egger’s regression test (*p* = 0.45) and Begg’s rank correlation test (*p* = 0.52).

##### Secondary Outcomes

**Fig. 3:**
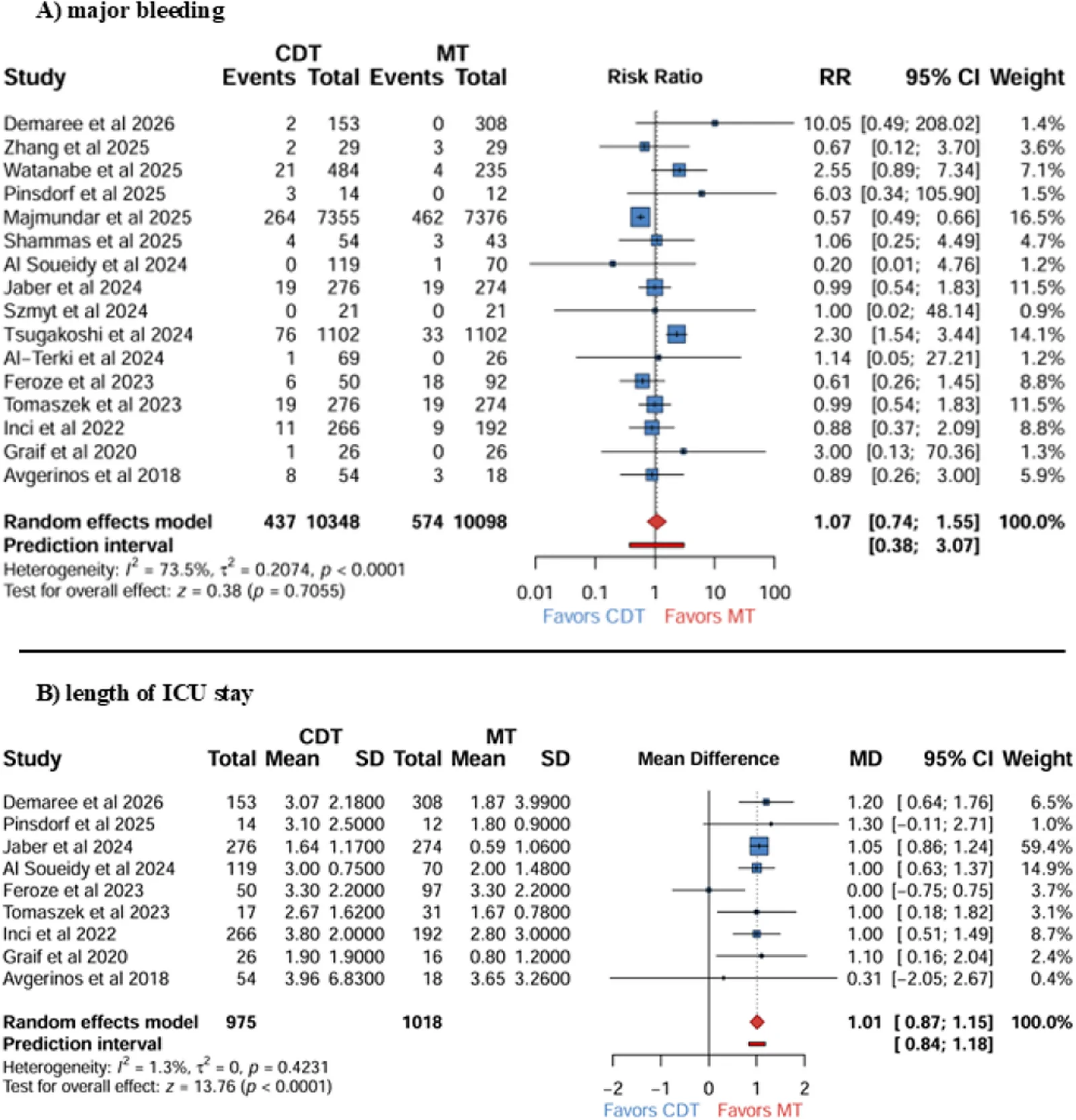
Forest plot for major bleeding and length of ICU stay. CDT, catheter directed thrombolysis; MT, mechanical thrombectomy; RR, risk ratio

CDT was associated with a similar risk of major bleeding (RR 1.07, 95% CI 0.74–1.55, *p* = 0.705, I^2^ = 73.5%, prediction interval 0.38–3.07, Fig. 3A) compared with MT. Although the sensitivity leave-one-out analysis confirmed these results for major bleeding without an obvious single-study effect, the statistical heterogeneity was resolved after exclusion of the *Majmundar et al. 2025 (*RR 1.23, 95% CI 0.86-1.76, I^2^ = 29.9%) *or Tsugakoshi et al. 2024 studies (*RR 0.89, 95% CI 0.64–1.22, I^2^ = 33.6%) from the main analysis (Figure S6).

**Fig. 4:**
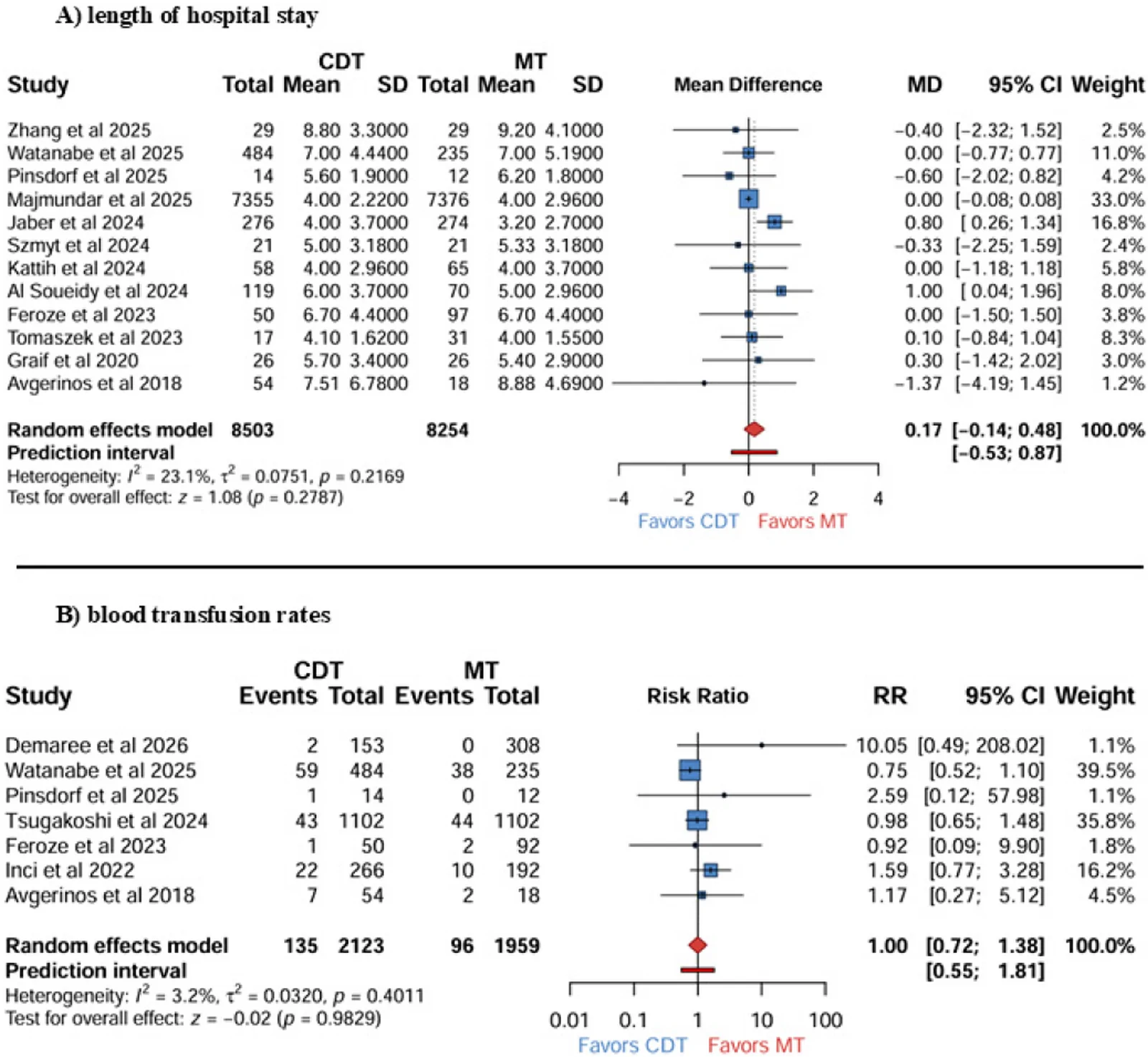
Forest plot for length of hospital stay and blood transfusion rates. CDT, catheter directed thrombolysis; MT, mechanical thrombectomy; RR, risk ratio

**Fig. 5:**
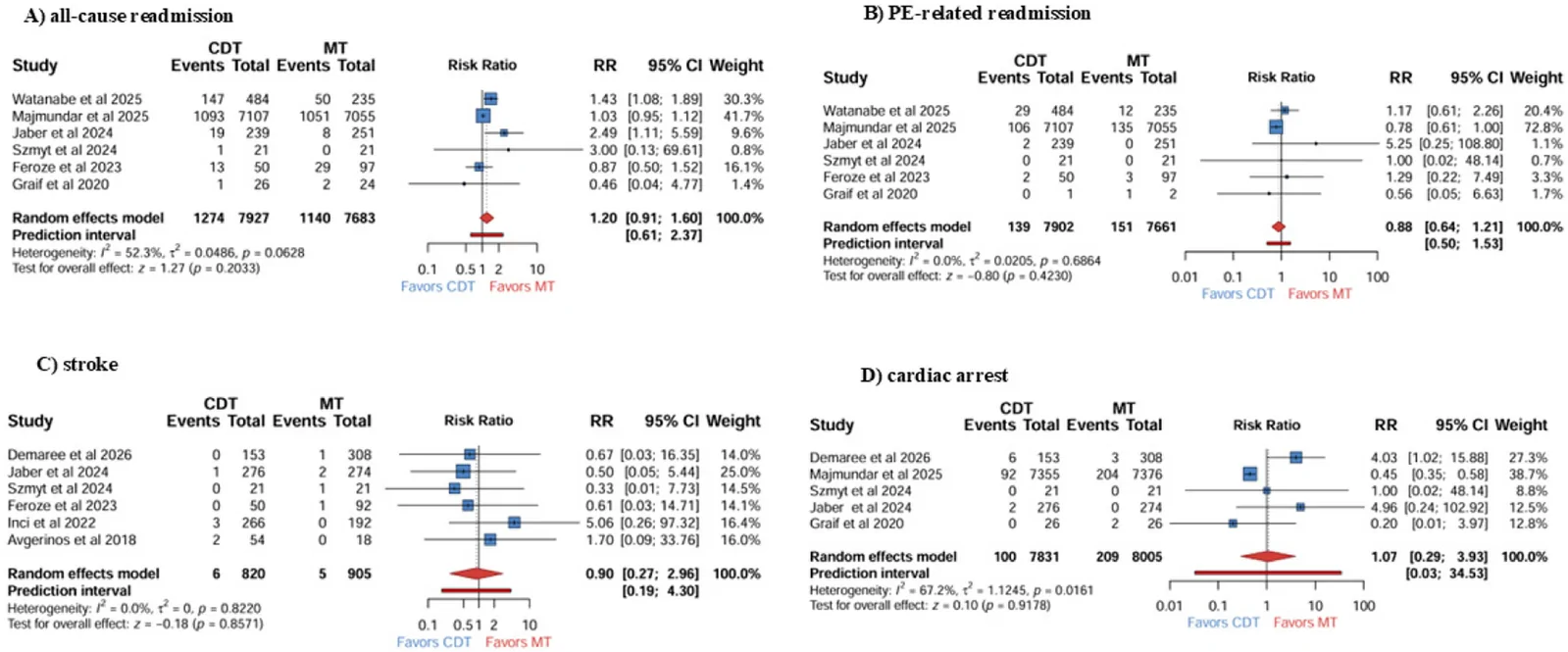
Forest plot for all-cause readmission, PE-related readmission, stroke, and cardiac arrest. CDT, catheter directed thrombolysis; MT, mechanical thrombectomy; RR, risk ratio

CDT was associated with a higher length of ICU stay compared with MT (MD 1.01 days, 95% CI 0.87–1.15, *p* < 0.0001, I^2^ = 1.3%, prediction interval 0.84–1.18, Fig. 3B**).** But there was no statistical difference between CDT and MT regarding the length of hospital stay, blood transfusion rates, risk of all-cause readmission, PE-related readmission, stroke, and cardiac arrest (Figs. 4 and 5).

## Discussion

In this meta-analysis of 18 studies including 24,090 patients with intermediate to high-risk PE who underwent catheter-based therapies, CDT and MT demonstrated comparable clinical outcomes. All-cause mortality was not statistically different between the two groups. While the subgroup analysis showed a lower risk of in-hospital all-cause mortality after the CDT compared with MT; but there was no difference regarding the other follow-up periods including ≤ 30 days, ≤ 1 year and 5 years. Major bleeding was also similar, while CDT was associated with a longer ICU stay. Most secondary outcomes, including readmission, blood transfusion, length of hospital stay, cardiac arrest, stroke, and procedural time, did not differ significantly between the two groups. Overall, our findings did not suggest that either strategy is superior to all patients.

These findings are consistent with the current shift in PE care toward more selective use of catheter-based therapies. Contemporary guidelines and consensus statements support catheter-based therapy in selected intermediate- or high-risk PE patients, especially when systemic thrombolysis is risky or when the patient is deteriorating, but they still do not strongly prefer CDT or MT because direct comparative evidence remains limited [8]. Earlier studies stated a safety and trend to more favorable outcomes after CDT for intermediate-to-high risk PE patients with an improvement in right/left ventricle (RV/LV) ratio [18, 27, 28]. Similarly, MT was shown as safe and effective method for high-risk PE patients [17]. Taken together, these studies have helped guide practice, but most were not designed to provide definitive head-to-head comparisons.

Our results are also in line with recent head-to-head literature. The PEERLESS randomized trial favored large-bore MT over CDT for its hierarchical composite endpoint and showed less clinical deterioration or bailout and lower post-procedural ICU use, but mortality and major bleeding were not clearly different [12]. In contrast, the National Readmission Database analysis by Majmundar et al. reported lower in-hospital mortality and major bleeding with CDT compared with MT, although this was a retrospective administrative dataset and is therefore vulnerable to coding and selection bias [13]. A recent meta-analysis by Afridi et al. also found broadly comparable outcomes between MT and CDT, with a possible advantage for MT in hospital stay [14]. Similarly, Demaree et al. reported no clear mortality difference in a single-center real-world comparison, while ICU use appeared lower with MT [15].

The mortality findings in our study need careful interpretation. The primary analysis did not reach statistical significance, while there was a lower risk of in-hospital mortality after CDT compared with MT. This signal should be interpreted cautiously and considered hypothesis-generating. In real practice, the patients selected for CDT and MT are often not identical. CDT may be preferred when controlled low-dose thrombolysis is acceptable, while MT may be chosen when thrombolysis is contraindicated or when rapid clot removal is needed [8, 12]. Additionally, some centers might prefer MT for patients after veno-arterial extracorporeal membrane oxygenation (VA ECMO), who are much sicker rather than CDT, but this data was not available across all studies and not extracted through this analysis [36]. The previous explanation might be the reason of higher in-hospital mortality after MT. Without patient-level data, it is difficult to separate the treatment effect from patient selection. Major bleeding was also not significantly different, which is reassuring, but this result should not be overread because bleeding depends on numerous factors, including thrombolytic dose, vascular access, anticoagulation, device size, and baseline bleeding risk. Additionally, a high heterogeneity was observed in the major bleeding outcome, so this result should be interpreted with caution.

One important factor not captured by the clinical endpoints in the present analysis is the acute hemodynamic effect of MT. MT rapidly reduces pulmonary artery (PA) pressure and RV afterload by physically removing thrombus from the pulmonary arterial tree, an effect that is evident within minutes to hours of the procedure. MT was associated with significant early reductions in the RV/LV ratio and PA systolic pressure, with improvements seen at 48 h in the FLARE and EXTRACT-PE trials [37, 38]. Similarly, the recent PEERLESS randomized trial comparing MT to CDT in intermediate–high-risk PE demonstrated that MT was associated with better immediate hemodynamic unloading as evidenced by a lower incidence of the composite of clinical deterioration, escalation of therapy or rescue reperfusion, but no significant difference in mortality or major bleeding between strategies [12]. By contrast, CDT usually requires a longer infusion (often 12–24 h or more) with more gradual hemodynamic effects [39, 40]. This temporal difference may partially explain the preferential selection of MT in patients with hemodynamic instability or impending RV failure and provides another potential source for selection bias in observational comparisons such as ours, where MT arms may systematically include sicker patients. Importantly, immediate hemodynamic improvement, as measured by reduction in RV/LV ratio, PA systolic pressure or the Miller index, is an outcome dimension not captured by the mortality-, bleeding- and resource-utilization-based endpoints of the present analysis, and should be prospectively evaluated in future randomized trials using standardized hemodynamic endpoints [8, 10].

The longer ICU stay with CDT is one of the most practical findings of this analysis. CDT often requires thrombolytic infusion and closer monitoring; ICU admission may reflect the treatment pathway rather than clinical instability alone. MT can sometimes provide immediate mechanical debulking and earlier step-down care, depending on center practice. From a health-system perspective, this finding is important because ICU availability, staffing, cost, and monitoring protocols can strongly influence which treatment is feasible. A strength of our study is that it includes the largest current comparative dataset, incorporates the PEERLESS trial and recent observational studies, and tests the findings with sensitivity, subgroup, meta-regression, and publication-bias analyses. These analyses strengthen the clinical relevance of our review, although the evidence remains evolving rather than definitive.

The CDT regimens used across the included studies were notably heterogeneous, as summarized in Table S2. Thrombolytic agents included alteplase, or urokinase, and delivery systems including standard multi-side hole catheters, ultrasound-assisted catheters (e.g., EKOS), and pharmacomechanical devices. Because both thrombolytic efficacy and bleeding risk are dose- and duration-dependent, this variability represents a clinically important source of heterogeneity that may influence both the efficacy and safety estimates derived from our pooled analysis. The PEERLESS post hoc analysis demonstrated that USAT achieved comparable clinical outcomes with significantly lower tPA doses and shorter treatment durations than standard CDT, suggesting that optimized low-dose protocols may be preferable [41]. However, standardized reporting of CDT dosing parameters remains inconsistent across the literature, limiting our ability to perform meaningful dose-stratified subgroup analyses.

## Limitations

This review has several limitations that affect how the findings should be interpreted. First, most included studies were retrospective cohorts, and only one study (PEERLESS trial) was a randomized controlled trial. The mortality signal should therefore be regarded as hypothesis-generating rather than confirmatory. Patients receiving CDT and MT may have differed in PE severity, clot burden, hemodynamic status, contraindications to thrombolysis, and center expertise, and residual confounding is very likely. To explore this, we performed subgroup analyses stratified by study design (randomized vs. observational) and by number of centers (single- vs. multicenter), as well as random-effects meta-regression; however, these analyses are limited by the small number of studies and cannot exclude confounding by indication. Second, clinical and methodological heterogeneity was substantial. PE-risk categories and outcome definitions were not uniform across studies, and the interventions themselves were heterogeneous, including different CDT thrombolytic dosing protocols, infusion durations, ultrasound-assisted systems, aspiration devices, and large-bore thrombectomy platforms. This is a known challenge in reviews of procedures and devices, where technology and operator experience evolve over time [33]. Third, a major limitation is that included studies enrolled mixed intermediate- and high-risk PE populations, and none reported outcomes stratified by PE risk category. Intermediate-low, intermediate-high, and high-risk PE differ in right ventricular dysfunction, hemodynamic compromise, and baseline mortality, and current guidelines recommend different reperfusion strategies for these strata. Pooling across risk categories may therefore mask clinically meaningful differences in the comparative effectiveness of CDT versus MT. For example, the immediate hemodynamic benefit of mechanical thrombectomy may be greatest in high-risk or hemodynamically borderline intermediate-high patients, and this signal would be diluted by pooling with intermediate-low patients. The comparative effectiveness of CDT versus MT within individual risk strata remains unknown. Fourth, the potential benefits of CDT may become apparent only during longer-term follow-up; specifically, improvements in pulmonary vascular remodeling, residual pulmonary hypertension, RV recovery, and exercise capacity, which may not be captured by the short-term outcomes evaluated in this meta-analysis. Additionally, the immediate hemodynamic effect of mechanical thrombectomy (rapid reduction in pulmonary artery pressure and RV afterload) is not captured by any of the clinical outcomes assessed and represents an unmeasured dimension of comparative effectiveness. Fifth, the substantial heterogeneity in CDT dosing protocols and device systems across the included studies represents a source of clinical heterogeneity that may affect the pooled estimates. We were unable to perform dose-stratified or device-stratified subgroup analyses due to inconsistent reporting of these parameters in the primary studies. Future trials should report standardized CDT dosing and device specifications to facilitate more precise comparative analyses.

## Future Directions

Future randomized trials and prospective registries should prespecify risk-stratified endpoints (intermediate-low, intermediate-high, and high-risk PE) to enable comparative-effectiveness analyses within clinically homogeneous strata.

## Conclusion

In patients with intermediate/high risk PE undergoing catheter-based therapy, CDT and MT showed no significant difference in the risk of mortality and major bleeding, while CDT was associated with longer ICU stay. But these findings should be interpreted as hypothesis-generating, given the predominantly retrospective evidence base and the inability to stratify by PE risk category. At present, treatment choice should remain individualized according to PE severity, bleeding risk, urgency of reperfusion, device availability, local expertise, and ICU resources. Future randomized comparative trials with standardized outcomes are needed to identify which patients benefit most from each strategy.

## Supplementary Information

Supplementary Material 1.

## Data Availability

The data is avaialable upon request.

## Abbreviations

BMI: Body mass index
CDT: Catheter-directed thrombolysis
CI: Confidence interval
DM: Diabetes mellitus
DOACs: Direct oral anticoagulants
ICU: Intensive care unit
LMWH: Low-molecular-weight heparin
MD: Mean difference
MT: Mechanical thrombectomy
PA: Pulmonary artery
PE: Pulmonary embolism
PRISMA: Preferred Reporting Items for Systematic Reviews and Meta-Analyses
REML: Restricted likelihood ratio
RoB 2.0: Risk of Bias tool
RRs: Risk ratios
USA: United States of America
VA ECMO: Veno-arterial extracorporeal membrane oxygenation
