# Supplementary material for: Outcomes with Catheter-Directed Thrombolysis versus Mechanical Thrombectomy for Patients with Intermediate/High Risk Pulmonary Embolism: A Meta-Analysis of 24,090 Patients
Source: Lung. 2026 Nov 10;204(1):94. doi: 10.1007/s00408-026-00950-7 (PMC13751712; doi:10.1007/s00408-026-00950-7)
Supplement: Supplementary file 1 [file 408_2026_950_MOESM1_ESM.docx]

**Outcomes with Catheter-Directed Thrombolysis Versus Mechanical Thrombectomy for Patients with Intermediate/High Risk Pulmonary Embolism: A Meta-Analysis of 24,090 patients.**

**Table S1. Data sources and detailed search strategy for each database.**

| Database | Search terms | Fields | date | Records |
| --- | --- | --- | --- | --- |
| PubMed | (catheter-directed thrombolysis OR CDT) AND (mechanical thrombectomy OR MT) AND (PE OR Pulmonary embolism) | All fields | 22nd June 2026 | 820 |
| Scopus | (catheter-directed thrombolysis OR CDT) AND (mechanical thrombectomy OR MT) AND (PE OR Pulmonary embolism) | Title, abstract, and keywords | 22nd June 2026 | 451 |
| WOS | (catheter-directed thrombolysis OR CDT) AND (mechanical thrombectomy OR MT) AND (PE OR Pulmonary embolism) | All fields | 22nd June 2026 | 350 |
| Cochrane CENTRAL | (catheter-directed thrombolysis OR CDT) AND (mechanical thrombectomy OR MT) AND (PE OR Pulmonary embolism) | Title, abstract, and keywords | 22nd June 2026 | 25 |

**Table S2. Characteristics of CDT and MT through included studies.**

| Study ID | CDT Technique | Guidance of CDT | EDMT Thrombolytic drug used | MT Technique |
| --- | --- | --- | --- | --- |
| Demaree et al 2026 | Unifuse or Cragg-McNamara | Ultrasound (US) | Recombinant plasminogen activator (rtPA) at 0.5-1 mg/h, while patients with two catheters received 1.0 mg/h divided between catheters | FlowTriever system |
| Shammas et al 2025 | EKOS (EkoSonic Endovascular System) | US | - | FlowTriever system |
| Zhang et al 2025 | Multiple-sided hole infusion catheters (Multi-Sideport) | Angiography | Urokinase (200,000–800,000 IU/day) | Penumbra Indigo aspiration thrombectomy system |
| Watanabe et al 2025 | - | - | - | - |
| Rosenzveig et al 2025 | - | - | - | - |
| Pinsdorf et al 2025 | EKOS | US | Alteplase at a rate of  1mg/h for 6**–**12 hours | FlowTriever system |
| Majmundar et al 2025 | - | - | - | - |
| Kattih et al 2024 | - | - | - | - |
| Al-Terki et al 2024 | EKOS | US and fluoroscopy | Alteplase 1 mg/h for 6 hours + unfractionated heparin with target PTT 60–80 s | Inari FlowTriever |
| Al Soueidy et al 2024 | EKOS | US | Low-dose tPA at 1 mg/hour per catheter, total dose 12–24 mg | Penumbra Indigo aspiration thrombectomy system |
| Jaber et al 2024 | EKOS | US | tPA infusion | FlowTriever system |
| Szmyt et al 2024 | Fountain 4F or 5F catheter | Angiography | Alteplase at a dose of 1 mg/h/catheter for 8 h with initial bolus of 2mg/catheter | Indigo catheter |
| Tsugakoshi et al 2024 | EKOS | - | - | FlowTriever system |
| Feroze et al 2023 | EKOS | - | - | Inari FlowTriever |
| Tomaszek et al 2023 | Simple straight pigtail or angled pigtail cathetes | Angiography | tPA drip rate of 0.5-1 mg/h | FlowTriever system |
| Inci et al 2022 | EKOS, UniFuse, or Cragg-McNamara infusion catheters | Fluoroscopy | - | FlowTriever |
| Graif et al 2020 | AP2 pigtail infusion catheter, UniFuse catheter, or EKOS ultrasound-assisted catheter | US | tPA infusion at rate 0.57 ± 0.17 mg/hour per catheter | FlowTriever large-bore aspiration system |
| Avgerinos et al 2018 | Cragg-McNamara or UniFuse or US-assisted thrombolysis with EKOS EkoSonic catheter | US | tPA on-table bolus: 2–4 mg followed by continuous infusion: 0.5–1.0 mg/hour | AngioVac, Arrow-Trerotola PTD, Penumbra Indigo aspiration thrombectomy system, or FlowTriever. |

**rtPA**, Recombinant plasminogen activator;

**Figure S1A.** ROB 2.0 for quality assessment of included RCT.

**Figure S1B.** ROBIN-I for quality assessment of observational studies.

**Figure S2.** Sensitivity analysis includes the studies that used the EKOS system for CDT for all-cause mortality.

**Figure S3.** Subgroup analysis for all-cause mortality based on follow-up.

**Figure S4.** Subgroup analysis for all-cause mortality based on study design.

**Figure S5.** Subgroup analysis for all-cause mortality based on centers.

**Figure S6.** Leave-one-out sensitivity analysis for major bleeding.
